# Piceatannol Affects Gastric Ulcers Induced by Indomethacin: Association of Antioxidant, Anti-Inflammatory, and Angiogenesis Mechanisms in Rats

**DOI:** 10.3390/life12030356

**Published:** 2022-02-28

**Authors:** Rasheed A. Shaik, Basma G. Eid

**Affiliations:** Department of Pharmacology and Toxicology, Faculty of Pharmacy, King Abdulaziz University, Jeddah 21589, Saudi Arabia; beid@kau.edu.sa

**Keywords:** indomethacin, piceatannol, gastric ulcers

## Abstract

One of the major aggressive factors that affect gastric injury is non-steroidal anti-inflammatory drugs (NSAIDs). Indomethacin (Indo) showed higher potentiality in gastric injury over conventional NSAIDs. Piceatannol (PIC) is a natural polyphenolic stilbene that possesses potent antioxidant and anti-inflammatory properties. The gastroprotective properties of PIC have been overlooked previously. Hence, we aim to study gastric injury induced by Indo and the protective action manifested by PIC, as well as to elucidate the likely underlying mechanisms of action in a rat model. The rats have been treated with vehicle, Indo alone, combined treatment with Indo, and PIC at (5 mg/kg or 10 mg/kg), respectively. The rats were also treated with Indo and omeprazole. In our study, we found that PIC at both 5 and 10 mg/kg doses was effective by averting the rise in ulcer and lesion indices, acid production, and histological variations persuaded by Indo. Mechanistically, PIC significantly reduced lipid peroxidation product (MDA), increased the GSH content, and enhanced SOD and CAT activity. In addition, PIC exhibits a distinct reduction in the levels of inflammatory parameters (Cox-2, IL-6, TNF-α, and NFκB). Contrastingly, PIC augmented both mucin and PGE2 content. Moreover, PIC fostered angiogenesis by increasing the expression of proangiogenic factors (VEGF, bFGF, and PDGF). Overall, the above results suggest PIC exhibits a potential protective effect against Indo-induced gastric ulcers by the antioxidant, anti-inflammatory, and angiogenic mechanisms.

## 1. Introduction

Major gastrointestinal diseases like gastric ulcers occur because of misproportioning between aggressive and protective factors in the stomach [1]. Belligerent aspects of the disease may involve nonsteroidal anti-inflammatory drugs (NSAIDs), smoking, mental stress, alcohol, and *H. pylori* infection. Protective factors may include growth factors, cell renewal, mucosal blood flow, nitric oxide (NO), prostaglandins, etc. [2]. NSAIDs are the most widely used analgesic and anti-inflammatory drugs [3]. NSAIDs are considered not only to induce gastric damage, but via various mechanisms, they delay the healing of ulcers [4]. Previously, indomethacin (Indo) has been shown to have a greater potentiality to cause gastric damage than conventional NSAIDs [5]. Previous Indo-induced ulcer studies showed that it inhibits the synthesis of prostaglandin-E2 (PGE2) or angiogenesis, facilitates the formation of free radicals, induces cyclooxygenase -2 (COX-2) expression, and induces cytokines responsible for pro-inflammatory actions [6,7]. Additionally, down-regulation of pro-angiogenic factors, like vascular endothelial growth factor (VEGF), platelet-derived growth factor (PDGF), and basic fibroblast growth factor (bFGF) by NSAIDs, delays healing of ulcers [8,9].

Piceatannol (PIC) is a natural polyphenolic stilbene found in peanuts, berries, sugar cane, white tea, grapes, and more. [10,11,12]. It has been reported that PIC has the potential for the management of chronic diseases [13]. Previous studies showed that PIC causes scavenging of free radicals and exerts potent antioxidant properties [14]. Furthermore, it can upregulate antioxidant-mediated defense mechanisms [15]. PIC also showed potent anti-inflammatory actions in both in vivo and in vitro studies [16]. The key mechanism behind its anti-inflammatory actions is the inhibition of COX-2, NF-κB, PGE2, and cytokines, such as TNF-α (Tumor Necrosis Factor-α), IL-6 (interleukin-6), interleukin-1β (IL1β), and NO [15,17,18]. All these properties could prove to be beneficial in preventing the development of gastric ulcers. The gastro-protective properties of PIC were previously overlooked. Hence, we aim to conduct our present study to explore Piceatannol’s mediated protective actions against Indo-induced gastric injury and elucidate the possible underlying mechanism(s) of action. We propose that PIC with its antioxidant and anti-inflammatory properties would protect against Indo-induced gastric ulceration in rats. This study could open new therapeutic potentials for natural products in the treatment of inflammatory diseases including peptic ulcers.

## 2. Materials and Methods

### 2.1. Use of Chemicals/Drugs

PIC, Indo, omeprazole, and carboxymethyl cellulose sodium (CMC-Na) were bought from (Sigma, St. Louis, MO, USA). Formalin, phosphate buffer, and all other necessary commercially available chemicals were selected based on the highest purity grade.

### 2.2. Study of Animals

In this study, adult male Wistar rats that weighed in the range of 200 to 220 g (10–12 weeks) were procured (source: Faculty of Pharmacy, KAU, Jeddah, KSA). The rats were housed in the animal house at controlled room temperature with relative humidity (50–55%), under a twelve-hour cycle (light and dark), at the Pharmacy faculty, KAU, department of pharmacology. The rats were allowed to acclimatize for one week to their environment and fed standard pelleted nutrients with spontaneous feeding of water. The whole experimental protocols have been carried out under the guidelines of the National Institute of Health (Animal care; NIH Pub no-8023; Revision no- 1978). Approval for this research has been obtained by the formed committee of research ethics of Pharmacy faculty, King Abdulaziz University (ref no- PH133-41).

### 2.3. Design of Experiments

Thirty rats were randomly divided into five groups (n = 6).

Group 1 (control): this group received the vehicle (CMC-Na 0.5% *w/v*, 10mL/ kg).

Group 2 (Indo): this group received only Indo (50 mg/kg) in a single shot, oral administration.

Group 3 (Indo + PIC 5 mg/kg), and group 4 (Indo + PIC 10 mg/kg): in these groups, PIC was administered orally, 5 mg/kg and 10 mg/kg, for seven days. On the seventh day, rats were given Indo 50 mg/kg orally followed 1hr later by the last dose of PIC.

Group 5 (Indo + Omeprazole): an oral dose of omeprazole (30 mg/kg) was given for seven days. On the seventh day, rats were given Indo 50 mg/kg orally followed 1 h later by the last dose of omeprazole. The antioxidant and anti-inflammatory properties of PIC have been tested at several dose levels in previous studies [19]. After four hours of Indo intoxication, rats in treatment groups were dissected.

### 2.4. Gastric Ulcer Induction

According to previous reports, Indo caused gastric ulceration [20]. The rats were deprived of the food supply with the liberty of water supply for 24 h on the sixth day. On the seventh day, Indo 50 mg/kg suspended in 0.5% CMC-Na was intragastrically administrated to all groups except the control group.

### 2.5. Measurement of Gastric Juice Volume and pH

After 4 h of Indo administration, ketamine (60 mg/kg) and xylazine (10 mg/kg) anesthesia was applied, and the abdomen was dissected. Thereafter, gastric pylorus was ligated followed by removal of the stomach portion and dissected via greater curvature. The stomach contents were immediately collected and transferred into a centrifugal tube followed by centrifugation for 5 min at 2500× *g* for eliminating debris before measuring the amounts of supernatants. A digital pH meter was used to measure the pH of the gastric juice (PHscan 40, Pocket PH tester, BANTE, China).

### 2.6. Morphological and Histological Examination

The rats’ stomachs were cut and rinsed with normal saline (0.9% NaCl). Digital photography was used for taking images. Through microscopy, the glandular mucus layers of the stomachs were then inspected to look for hemorrhagic lesions. Each lesion’s length was measured in millimeters (mm). The ulcer index was calculated using the equation below:

Ulcer index = 10 ÷ x

x = the total ulcerated/mucosal area [21].

The samples (tissues) were fixed in formalin to produce paraffin blocks before being sliced as 4 µm thick sections with a sliding microtome. Staining was done by eosin and hematoxylin and inspected under the light microscope (Nikon, Eclipse) for routine histological examination [22]. A histopathologist evaluated the pathological alterations and other scoring according to a previous study [23].

### 2.7. Mucin Protein Assessment

By using a Rat MUC1 ELISA kit (cat ≠ NBP2-74991, Novus Biologicals, CO, USA), mucin protein was assessed based on the sandwich technique. Stomach tissues were chopped into fine particles and, to remove excess blood, thoroughly washed in ice-cold phosphate-buffered saline (PBS). Tissue pieces were taken for measuring their weight and homogenization was performed in PBS with glass homogenizer in ice as *w/v* of tissue weight (g): PBS (ml) = 1:9. The homogenates were centrifuged at 5000× *g* for 5 min for obtaining supernatant. Each well received 100 μL of standard or sample and was incubated for 90 min/37 °C. The supernatant was discarded and 100 μL of detection antibody was incubated for 1 h/37 °C before being aspirated and washed as thrice. After that, 100 μL of HRP conjugate was added and incubated for 30 min/37 °C; aspirating followed by washing five times. A total of 90 μL of substrate reagent was incubated for 15 min/37 °C followed by adding a 50 μL stop solution and the optical density (OD) at 450 nm was measured immediately.

### 2.8. Assessment of Oxidative Stress Markers

MDA was determined using a biochemical kit (Cat. no MD 2529, Bio diagnostic, Giza, Egypt). Thiobarbituric acid (TBA) reacted with malondialdehyde (MDA) for half an hour in an acidic medium at 95 °C to form thiobarbituric acid reactive product. The absorbance of the pink product that resulted was measured at 534 nm. The product (thiobarbituric acid reactive product) was formed after 30 min of TBA (thiobarbituric acid) reaction with MDA (malondialdehyde) in an acidic medium at 95 °C [23]. Glutathione (GSH) was measured by using the biochemical kit (Cat. No K264, Biovision, Milpitas, CA, USA). SOD enzymatic activity was measured by a colorimetric method using a biochemical kit (Cat. No. SD 2521, Biodiagnostic, Giza, Egypt) [24] and CAT activity was measured biochemically (Cat. no CA 25 17, Bio diagnostic, Giza, Egypt) [25].

### 2.9. Prostaglandin E2 (PGE2) Assessment

PGE2 activity evaluation in gastric tissue homogenates was carried out by Rat Prostaglandin E2 (PGE2) ELISA Kit (cat # CSB-E07967r, Cusabio, Houston, TX, USA), based on competitive inhibition enzyme immunoassay technique.

### 2.10. Immunohistochemical Assessment of Cox-2, IL-6, TNF-α, NF-κβ, and VEGFA

For immunohistochemical studies, sample sections were incubated with primary antibodies: COX-2 (cat# ab179800 Abcam^®^, Cambridge, UK), IL-6 (Cat# ab9324, Abcam^®^, Cambridge, UK), TNFα (Cat. No.: ab220210, Abcam^®^, Cambridge, UK), NFκB (Cat# ab194726 Abcam^®^, Cambridge, UK), and VEGFA (Cat# ab231260 Abcam^®^, Cambridge, UK) for 12 h/4 °C. After a TBS rinse, samples were incubated in anti-mouse or anti-rabbit biotinylated secondary antibody, depending on the primary antibody reactivity. (Cell & Tissue Staining Kit, Cat. No.: CTS002 & CTS005, R&D systems, Minneapolis, MN, USA). Images had quantified by image analysis software (Image J, 1.46a, NIH, Bethesda, MD, USA) with a minimum of three sections per rat. It is noteworthy to report that only nuclear reactivity was considered when assessing the expression of NFκB.

### 2.11. Quantitative Reverse Transcriptase PCR (qRT-PCR)

The GeneJET Kit was used to harvest total RNA (Thermo Fisher Scientific Inc. Germany, Cat no. K0732). Then one step qRT-PCR was performed by using SensiFAST™ SYBR^®^ Hi-ROX One-Step Kit (Cat no. PI-50217V). The primers that have been used as per sequences are mentioned in Table 1. After qRT-PCR amplification, the mRNA fold change was calculated by normalizing the gene of interest with the housekeeping gene and presented as 2^−ΔΔCt.^

### 2.12. Statistical Analysis

All data are presented as means ± SD. Ulcer index data were analyzed by Kruskal–Wallis test followed by Dunn multiple comparison tests. The other data were analyzed by one-way analysis of variance (ANOVA), followed by Tukey’s multiple comparison test to detect significant differences. A value of *p* < 0.05 was considered statistically significant. GraphPad Prism software version 8 (GraphPad Software, Inc., La Jolla, CA, USA) was used for statistical analysis.

## 3. Results

### 3.1. Effects of PIC on Gastric Juice Volume and pH

**Figure 1:**
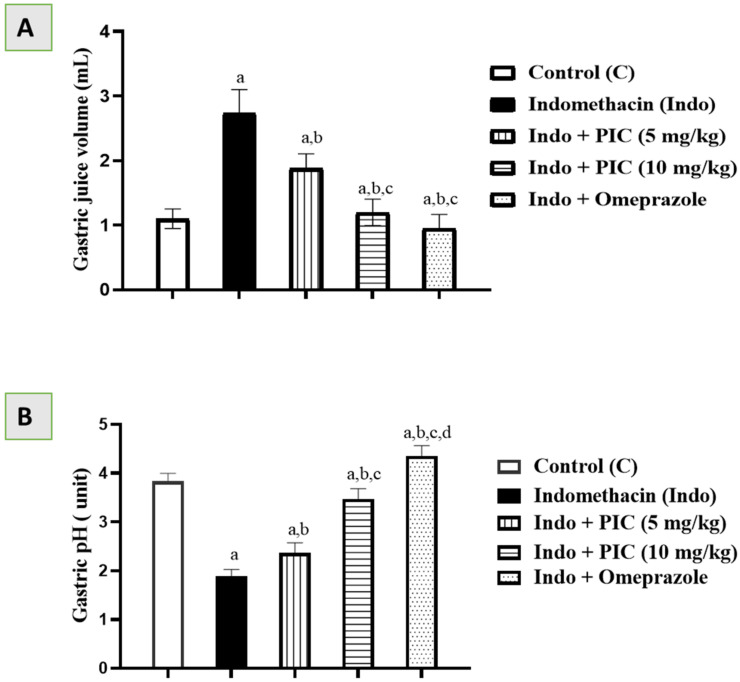
Effects of PIC on gastric juice volume and pH. (**A**) Gastric juice volume for different groups. (**B**) Gastric pH for different groups. Data are presented as Mean ± SD (n = 6). a: Significantly disparate with control (*p* < 0.05); b: significantly disparate with Indo (*p* < 0.05); c: significantly disparate with Indo + PIC (5 mg/kg) (*p* < 0.05); and d: significantly disparate with Indo + PIC (10 mg/kg) (*p* < 0.05).

Gastric juice volume and pH in the control group were 1.10 mL and 3.83 units. The Indo group showed (Figure 1A) a significant rise in gastric juice volume (2.74 mL) and a substantial (Figure 1B) reduction in gastric juice pH (1.9 units). PIC at both 5 and 10 mg/kg doses considerably reduced gastric juice quantity (1.88 mL & 1.2 mL) and elevated gastric juice pH (2.37 & 3.47 units) (Figure 1A,B). Omeprazole significantly reduced gastric juice volume (0.95 mL) and elevated gastric juice pH (4.35 units) markedly (Figure 1A,B). Indo + PIC 10 mg/kg and Indo + Omeprazole showed an almost similar significant effect (Figure 1A,B).

### 3.2. Effect of PIC Pretreatment on Stomach Morphology and Histopathology

**Figure 2:**
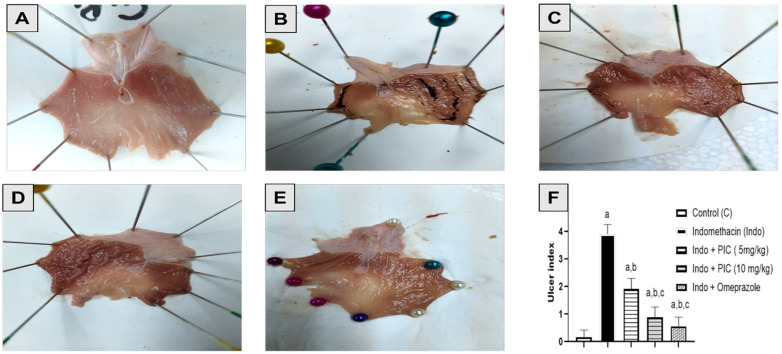
Macroscopic photographs of rat’s Stomachs. (**A**) The Control group showed gastric mucosa with no lesion or redness. (**B**) Rats with treated Indo had a severely hemorrhagic ulcerated mucous surface. (**C**) Indo + PIC (5 mg/kg) showing superficial injuries. (**D**) Indo + PIC (10 mg/kg) showing minor injuries with normal mucosa. (**E**) Indo + Omeprazole effectively normalizes the damaged mucosal layer and shows no redness or damage. (**F**) Ulcer index. The data are presented as mean ± SD. a: Significantly disparate with control (*p* < 0.05); b: significantly disparate with Indo (*p* < 0.05) and c: significantly disparate with Indo + PIC (5 mg/kg) (*p* < 0.05).

The mucous lining in the stomach of the control group was normal and null injury was noted (Figure 2A). Nevertheless, the Indo group exhibited bloody streaks injuries ranging from 0.5–5 mm in size (Figure 2B), as measured by the dramatic rise in ulcer index (Figure 2F). Indo+ PIC (5 mg/kg) exhibited bloody streaks, although to a smaller extent as compared with the Indo group (Figure 2C). Rats given Indo+ PIC (10 mg/kg) showed superficial blood vessel blockage suggesting minor injuries, with a marked decline in ulcer index than the Indo group (Figure 2D). Omeprazole treatment safeguarded the layer of gastric mucosa, with no marked variation in ulcer index compared to control (Figure 2E,F).

**Figure 3:**
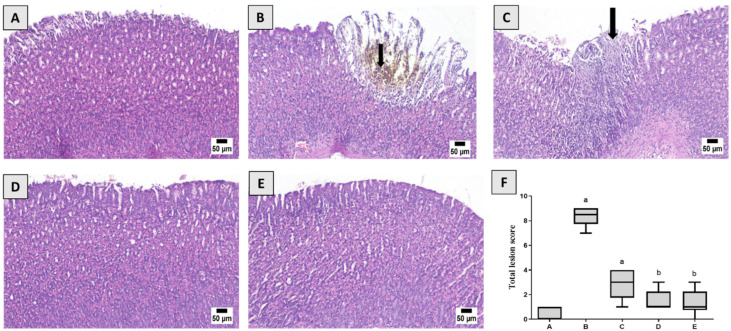
Histological examination. (**A**) Stomach from control group showing normal gastric mucosa. (**B**) Stomach from the Indo-treated group showing ulcerative mucosal surface associated with deposition of hemosiderin pigment (arrow). (**C**) Stomach from Indo + PIC (5 mg/kg) showing focal necrosis of mucosal surface (arrow). (**D**) Stomach from Indo + PIC (10 mg/kg) showed apparently normal mucosa. (**E**) Stomach from Indo + omeprazole showed normal stomach mucosa. (**F**) Total lesion score. The data are presented as mean ± SD. a: Significantly disparate with control (*p* < 0.05); b: significantly disparate with Indo (*p* < 0.05).

As shown in Figure 3, in contrast, to the control group, serious histopathological alterations were detected in the Indo group. Multifocal ulcerative areas were detected in the glandular mucosa which was characterized by desquamation of the epithelial lining admixed with hemorrhages and accumulation of necrotic tissue and hemosiderin pigment. Numerous sections showed excessive inflammatory cells infiltration in the mucosa and submucosal layers. The submucosa layer revealed dispersion of the connective tissue with abundant edema, congested blood vessels, and inflammatory cells infiltration. The examination of Indo+ PIC (5 mg/kg) revealed a few changes characterized by mild epithelial sloughing with numerous inflammatory cells infiltration in the mucosa and submucosal layer. Microscopic examination of Indo + PIC (10 mg/kg) showed the mostly normal histological structure of glandular mucosa and submucosa with minor injuries in several examined sections. A normal glandular stomach was observed in omeprazole pretreated rats as well. The statistical evaluation of total histopathological alterations exhibited a significant rise in the Indo group in comparison to control or treated groups. The absence of significant difference was observed between Indo + PIC 10 mg/kg and Indo + Omeprazole (Figure 3D,E).

### 3.3. PIC Affects the Mucin Content

**Figure 4:**
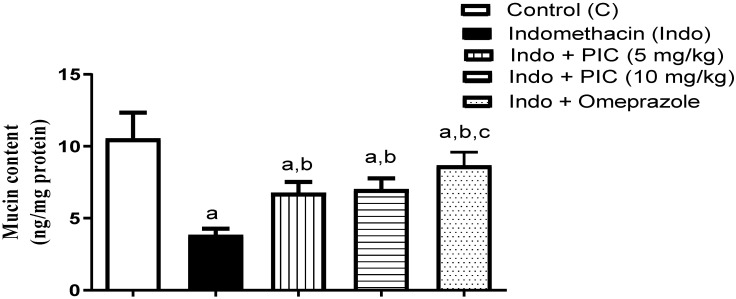
The mucin level in gastric tissue after PIC treatment with Indo. Data are presented as Mean ± SD (n = 6). a: Significantly disparate with control (*p* < 0.05). b: Significantly disparate with Indo (*p* < 0.05); c: Significantly disparate with Indo + PIC (5 mg/kg) (*p* < 0.05).

Our results indicated that the Indo group markedly decreased mucin level by 63.66% compared to the control, whereas the decline was significantly reversed by PIC (5 & 10 mg/kg) and omeprazole treatments by 1.77-, 1.83-, and 2.26-fold, respectively (Figure 4).

### 3.4. PIC Affects the Oxidative Stress Denoting Markers

PIC mediated antioxidant actions had been evaluated by measuring MDA level, GSH content, and SOD and CAT activity in gastric homogenate. As revealed in Table 2, Indo caused MDA accumulation, GSH depletion, and CAT and SOD exhaustion by 470.83%, 70.45%, 55.31 %, and 45.45%, respectively, compared to control group. PIC (5 or 10 mg/kg) and omeprazole treatment significantly reduced the MDA level by 31.38%, 43.55%, and 58.64%, with respect to the Indo group (Table 2). A substantial rise in gastric GSH content was observed in PIC (5 or 10 mg/kg) and omeprazole groups by 46.15%, 61.53%, and 76.92%, respectively. Rats pretreated with PIC (5 & 10 mg/kg) and omeprazole showed a significant increase in SOD activity by 39%, 65.75%, and 88.17%, respectively. Moreover, a marked rise in CAT activity has been reported in PIC (5 or 10 mg/kg) and omeprazole groups by 41.66%, 60.41%, and 47.91 %, respectively, when compared with the ulcer control group (Table 2).

### 3.5. The Level of Gastric PGE2 Content

**Figure 5:**
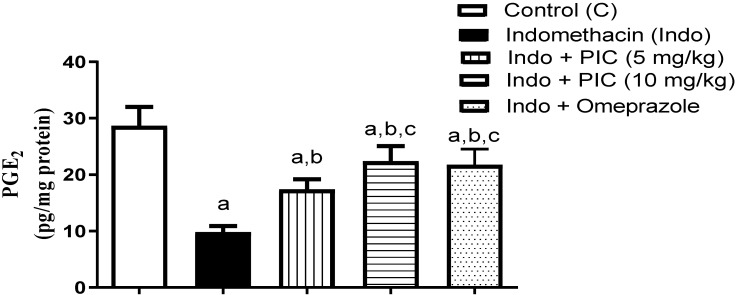
The level of gastric PGE2 content after PIC treatment with Indo. Data are presented as Mean ± SD (n = 6). a: Significantly disparate with control (*p* < 0.05). b: Significantly disparate with Indo (*p* < 0.05); c: Significantly disparate with Indo + PIC (5 mg/kg) (*p* < 0.05).

Administration of Indo showed a significant decrease in the gastroprotective PGE2 level; 65.83% in comparison with the control. However, pretreatment with PIC (5mg and 10mg/kg) or omeprazole considerably augmented the PGE2 level by 77.58%, 128.86%, and 122.41%, compared to the Indo group, respectively. (Figure 5).

### 3.6. Immunohistochemical Assessment of Cox-2, IL-6, TNF-α, NF-κβ, and VEGFA

**Figure 6:**
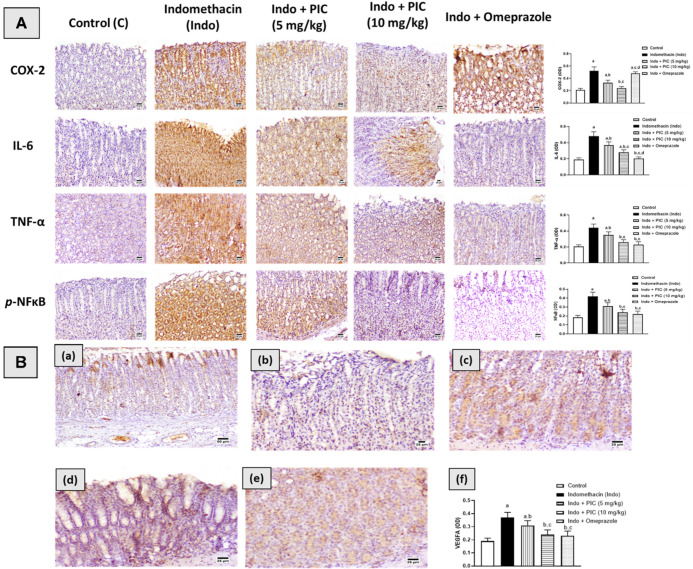
Immunohistochemistry examination of Inflammatory markers and proangiogenic factor. (**A**). Immunohistochemistry examination of Cox-2, IL-6, TNF-α, and p-NF-κβ expression in stomach sections. (**B**): Immunohistology photomicrographs of stomach sections demonstrating the effect of PCI on Indo-induced alteration in VEGFA. (**a**) The Control group shows an intense expression of VEGFA. (**b**) The Indo-treated group shows a decreased expression of VEGFA. (**c**) Indo + PIC (5 mg/kg) group shows a moderate expression of VEGFA. (**d**) Indo + PIC (10 mg/kg) group shows an intense expression of VEGFA. (**e**). Indo + Omeprazole group shows increase expression of VEGFA. (**f**) Quantitative image analysis for VEGF immunohistochemical staining, expressed as optical density (OD). The data are presented as mean ± SD. a: Significantly disparate with control (*p* < 0.05); b: significantly disparate with Indo (*p* < 0.05); c: significantly disparate with Indo + PIC (5 mg/kg) (*p* < 0.05); and d: significantly disparate with Indo + PIC (10 mg/kg) (*p* < 0.05).

Administration of Indo significantly increased the expression of Cox-2, IL-6, TNF-α, and NF-κβ by 147.62%, 152.63%, 114.63%, and 127%, respectively, compared to the control group. Pretreatment with PIC 5 mg/kg resulted in respective decreases in their expression of 36.53%, 23%, 20.45%, and 26.19%, compared to the Indo group. The rats treated with PIC 10 mg/kg and Indo exhibited decreases in their expression of 53.84% 41.7%, 41%, and 43%, respectively. In addition, pretreatment with omeprazole showed an increased expression of Cox-2 like Indo. Furthermore, omeprazole-induced decreased expression of IL-6, TNF-α, and NF-κβ by 58%, 48%, and 47.7%, respectively, compared to the Indo group. However, VEGFA expression was markedly reduced by 43% after Indo treatment compared to the control group. The PIC (5 or 10 mg/kg) and omeprazole 30 mg/kg groups showed significant increases in VEGFA expression, 55%, 70%, and 60%, respectively, in comparison with the Indo group. The immunohistochemical findings have been presented in Figure 6A,B.

### 3.7. Effect of PIC on mRNA Expression Levels of bFGF and PDGF Marker Genes

**Figure 7:**
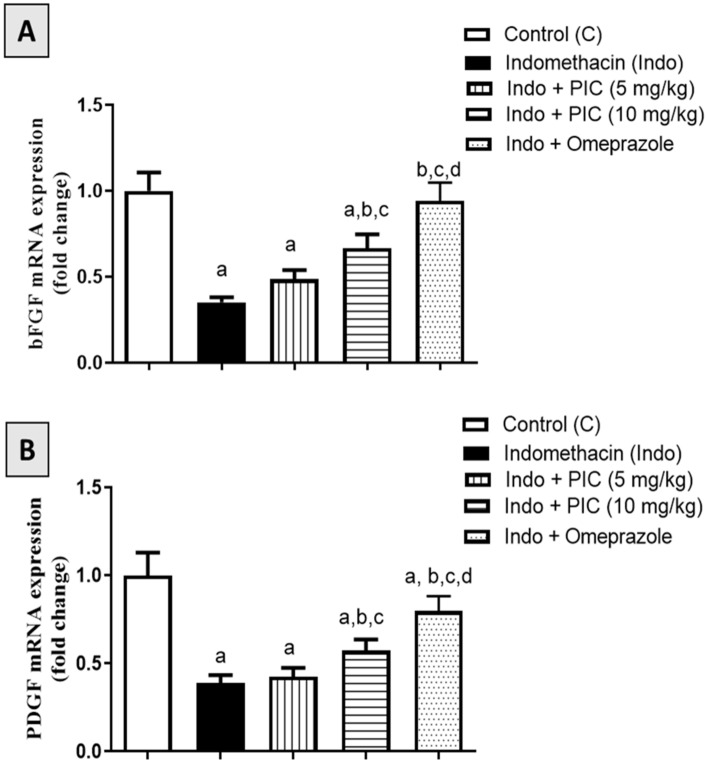
Effect of PIC pretreatment on (**A**) bFGF and (**B**) PDGF mRNA expression levels in gastric ulcer induced by Indo. Data are presented as Mean ± SD (n = 6). a: Significantly disparate with control (*p* < 0.05). b: Significantly disparate with Indo (*p* < 0.05); c: Significantly disparate with Indo + PIC (5 mg/kg) (*p* < 0.05); d: Significantly disparate with Indo + PIC (10 mg/kg) (*p* < 0.05).

Rats treated with Indo showed a significant decrease in the bFGF mRNA expression by 64.77% compared to the control (Figure 7A). PIC (5 or 10 mg/kg) prevented the downregulation by 38.23% and 89.48%, respectively. In addition, omeprazole also prevented the decrease in their expression by 167.6% compared to the Indo group. Moreover, Indo-treated rats showed significant downregulation in PDGF expression by 60.96% compared to the control values (Figure 7B). Pretreatment with PIC (5 or 10 mg/kg) and omeprazole 30 mg/kg prevented the downregulation by 9.23%, 46.92%, and 104.6%, respectively (Figure 7B).

## 4. Discussion

One of the most common diseases suffered by people in their lifetime is a gastric ulcer, which is driven by several mucosal factors [26,27]. A study revealed several mechanisms of gastric mucosal defense systems, like mucus bicarbonate phospholipid barrier, epithelial cells derived mucus/bicarbonate production, mucosal cell renewal, etc., are involved. The study also revealed the mechanisms of gastric mucosal damage [28]. NSAIDs are associated with gastric ulcer development and other adverse effects on the gastrointestinal environment [29]. Additionally, a previous study showed that Indo causes gastric injury in rats by inducing the reactive oxygen species (ROS) level [30,31]. In contrast, PIC is a known natural polyphenolic stilbene [32] and a resveratrol metabolite that exerts potent antioxidant and cytoprotective roles, and ROS chelation [33,34]. A previous study showed that gastric injury induced by Indo in rats can be reversed by L-arginine and Chinese sumac [35,36]. PIC-mediated reversal of gastric injury induced by the Indo study has been overlooked. Hence, this study aimed to investigate this gap.

Our experimental results showed that the Indo group had a severe hemorrhagic ulcerative mucosal lesion, associated with intense expression of COX-2, IL6, TNFα, NF-κB, and lower expression of VEGFA, compared to the control group. In addition, it also showed higher gastric juice volume with lower gastric pH. The mucin level in the Indo-treated group was also reduced. This was also reported in a previous study [37]. Additionally, the PGE2 level and the expression of bFGF and PDGF were also reduced. Compared to the control, the Indo group also had an induced MDA level, reduced GSH content, and depleted SOD and CAT activity that denotes the upraise of ROS induction pathway. A similar previous study also supported these findings [38]. Cumulatively the Indo group results indicate a severe gastric injury and delayed healing. One previous rat study showed that Indo induced the COX-2 expression [39]; simultaneously, another previous study revealed that Indo has been shown to induce the IL6/TNFα level and inhibited the PGE2 level [40]. While in normal gastric mucosa COX-1 is the predominant COX isoenzyme, there is increasing evidence that detectable amounts of COX-2 mRNA and protein are expressed both constitutively and inducible in gastric mucosa [41]. Several reports indicated an increased expression of COX-2 in damaged gastric mucosa [42,43,44]. In particular, our data gain support by several studies reporting an association between Indo-induced gastric injury and enhanced expression of COX-2 [6,39]. Up-regulation of COX-2 expression following inhibition of COX-1 may represent a compensatory response to inhibition of PG biosynthesis and contribute to the maintenance of the mucosal integrity under such conditions. This has been explained on the basis that gastric hypermotility is critical for the occurrence of initial damage under PG deficiency caused by NSAIDs [45,46]. Controversial findings regarding PDGF and FGF2 in Indo-treated rats were also reported [47,48].

In contrast, Indo+ PIC (5 mg/kg) showed superficial injuries with focal necrosis, associated with moderate expression of COX-2, IL6, TNFα, NF-κB, and VEGFA compared to the Indo group. The mucin level in this group showed a significant increase compared to the Indo group. Similar results were also found in the case of the PGE2 level. Compared to the Indo group, it also had a significant reduction in MDA level and slight induction of GSH content, and an increase in the SOD and CAT activity. It also significantly increases the bFGF and promotes cellular growth. Interestingly, pretreatment of PIC (5 mg/kg) did not significantly affect PDGF mRNA expression.

These results have been observed in the case of rats treated with Indo + PIC (10 mg/kg). It showed that treatment reversed the gastric injuries induced by Indo and exhibit normal mucosa. Additionally, it had lower expression of COX-2, IL-6, TNF-α, and NFκB, and was associated with a significant reduction in gastric volumes and induced level of gastric pH. Surprisingly it did not induce the mucin level compared to Indo + PIC (5 mg/kg); however, it slightly induced the PGE2 level. Furthermore, induction of bFGF and PDGF mRNA were observed in the PIC (10 mg/kg) pretreated group. It also showed a significant reduction in MDA, induction of GSH, and enhancement of SOD and CAT activity. The previous studies also revealed that PIC has a therapeutic role especially in metabolic disorders like antioxidant (through lowering ROS), anti-inflammatory (through lowering IL-6, TNF-α), and by suppressing the cytokine signaling pathways [16,32]. Another study showed that PIC reduced 12-O-tetradecanoylphorbol-13-acetate (TPA) induced COX-2 expression by blocking the activity of NF-κB [19]. However, no direct study regarding the effect of PIC on gastric ulcers has been found yet.

The most effective results have been observed in the Indo + omeprazole group. It showed the damage of stomach mucosa was normalized by obtaining intense expression of COX-2 and VEGFA, and weak expression of other cytokines (IL-6, TNF-α). Additionally, the omeprazole pretreated group had a significantly lower level of gastric volumes and a significantly increased amount of gastric pH compared to other groups. Interestingly, omeprazole induces mucin content slightly more compared to other groups with a slight reduction in PGE-2 content compared to Indo + PIC (10 mg/kg). Moreover, it had a significant induction of bFGF in line with previous findings [38]. Omeprazole also increased the PDGF mRNA expression. The antioxidant results of the omeprazole pretreated group suggested a reduction in the level of ROS from the significant reduction in MDA level and significant induction of GSH content and SOD activity. Surprisingly, it slightly reduced CAT activity compared to Indo + PIC (10 mg/kg). A previous study showed that omeprazole assists in ulcer healing by inducing COX-2 and PGE2 levels. The authors of this study suggested that the gastrin-dependent pathway is accountable for increased expression of COX-2 and elevated levels of PGE2 [49,50]. In other words, NF-κB-dependent induction of COX-2 has minor or no effect on the anti-ulcerogenic activity of omeprazole. This is because NSAID-induced gastric damage is known to include acid-dependent lesions, and because omeprazole inhibits gastric acid secretion through suppression of H+/K+ ATPase in the parietal cells [51]. Moreover, a previous study also showed an omeprazole-like proton pump inhibitor (PPI) [52] induced the VEGFA expression [53]. Similar findings regarding MDA, GSH, SOD, and CAT levels were also found in previous rat studies [54].

## 5. Conclusions

From the above discussion, it may be concluded that treatment with PIC may normalize the gastric injuries induced by Indo. This finding could lead to strategies for treating gastric ulcers by a surplus of new therapy over conventional treatment.

## Figures and Tables

**Table 1:** Primer sequence of target genes.

	Primer (5′ to 3′)
*PDGF*	F: ATTGGCAATGAGCGGTTCCGC
	R: CTCCTGCTTGCTGATCCACATC
*BFGF*	F: TGGCATTCTCAGGTTCTGGCCATT
	R: TGGCATTCTCAGGTTCTGGCCATT
*β-Actin*	F: TCCGTCGCCGGTCCACACCC
	R: TCACCAACTGGGACGATATG

**Table 2:** PIC effects on oxidative stress biomarkers.

	MDA (nmol/mg Protein)	GSH (µg/mg Protein)	SOD (U/mg Protein)	CAT (U/mg Protein)
Control (C)	0.72 ± 0.09	0.44 ± 0.05	39.92 ± 4.21	0.88 ± 0.10
Indomethacin (Indo)	4.11 ^a^ ± 0.45	0.13 ^a^ ± 0.02	17.84 ^a^ ± 2.10	0.48 ^a^ ± 0.06
Indo + PIC (5 mg/kg)	2.82 ^a,b^ ± 0.31	0.19 ^a,b^ ± 0.02	24.80 ^a,b^ ± 2.86	0.68 ^a,b^ ± 0.08
Indo + PIC (10 mg/kg)	2.32 ^a,b,c^ ± 0.28	0.21^a,b^ ± 0.02	29.57 ^a,b^± 3.31	0.77 ^b^ ± 0.09
Indo + Omeprazole	1.70 ^a,b,c,d^ + 0.19	0.23 ^a,b^ ± 0.03	33.57 ^a,b,c^ ± 3.94	0.71 ^a,b^ ± 0.09

Data are presented as Mean ± SD (n = 6). ^a^: Significantly disparate with control (*p* < 0.05). ^b^: Significantly disparate with Indo (*p* < 0.05). ^c^: Significantly disparate with Indo + PIC (5 mg/kg) (*p* < 0.05). ^d^: Significantly disparate with Indo + PIC (10 mg/kg) (*p* < 0.05).

## Data Availability

Data are contained within the article.
